# The First Southern Hemisphere Occurrence of the Extinct Cretaceous Sclerorhynchoid Sawfish *Ptychotrygon* (Chondrichthyes, Batoidea), With a Review of *Ptychotrygon* Taxonomy

**DOI:** 10.1080/02724634.2022.2162411

**Published:** 2023-02-09

**Authors:** Arnaud Begat, Jürgen Kriwet, Javier N. Gelfo, Soledad Gouiric Cavalli, Julia A. Schultz, Thomas Martin

**Affiliations:** 1 Department of Palaeontology, Faculty of Earth Sciences, Geography and Astronomy, University of Vienna, Josef-Holaubek-Platz 2, Vienna, 1090, Austria; 2 Vienna Doctoral School of Ecology and Evolution (VDSEE), University of Vienna, Djerassiplatz 1, Vienna, 1030, Austria; 3 CONICET, Divisiόn Paleontología Vertebrados, Museo de La Plata, Paseo del Bosque S/N, La Plata Buenos Aires, B1900FWA, Argentina; 4 Institute for Geosciences, Section Paleontology, Rheinische Friedrich-Wilhelms-Universität Bonn, Nußallee 8, Bonn, 53115, Germany

## Abstract

A new extinct sclerorhynchoid sawfish, *Ptychotrygon ameghinorum* sp. nov., is presented here based on abundant isolated teeth and some dermal denticles, which were recovered from the Mata Amarilla Formation, belonging to the lower Upper Cretaceous of the Santa Cruz Province in the Austral Basin of Patagonia, Argentina. This new species is the first *Ptychotrygon* occurrence in the southern hemisphere, which so far only has been reported from northern hemisphere deposits (Europe, North Africa, and North America). The presence of *P. ameghinorum* sp. nov. in these southern high-latitude deposits of Patagonia, Argentina, extends the geographic range of *Ptychotrygon* considerably southwards. This distribution pattern in the “middle” Cretaceous seems to correlate with the South Atlantic opening at the end of the Albian. The presence of lateral cephalic dermal denticles and the simultaneous absence of rostral denticles in the abundant fossil material support the view that *Ptychotrygon* did not develop such rostral structures. A reinvestigation of all known species assigned to *Ptychotrygon* reveals that *P. ellae* is a junior synonym of *P. boothi, P. benningensis* belongs to *Texatrygon, P. rugosum* belongs to *Asflapristis*, and *P. clementsi* represents an unidentifiable species (*Ptychotrygon*? sp.). The stratigraphic distribution demonstrates that *Ptychotrygon* might have originated in the Albian in south-western Europe and subsequently dispersed to obtain its widest distribution during the Cenomanian. In the Coniacian, a steep diversity decline is recognizable with a subsequent distribution shift from Europe to North America.

## Introduction

Sclerorhynchoidei represent an extinct group of skates (Batomorphii, Rajiformes) that seemingly was successful during most of the Cretaceous period in terms of species richness and distribution (Kriwet & Benton, 2004). This clade is characterized by a hypertrophied rostral cartilage bearing lateral rostral denticles with a specific replacement mode and modified dermal denticles on the lateral cephalic region (Cappetta, 1980). An elongated rostrum evolved several times independently in various batoid groups and thus bears a strong functional signal. Sclerorhynchoids appeared in the fossil record during the Barremian and finally went extinct during the K/Pg boundary event (Kriwet, 1999b; Cappetta, 2012). This group generally is divided into three families. The most diversified family is the Sclerorhynchidae comprising 22 genera, while the other well-defined family, the Ptychotrygonidae, is represented by five genera (*Archingeayia* included) (e.g., Cappetta, 2012). Recently a third family, the Onchopristidae, was defined including so far a single genus (*Onchopristis;* Villalobos-Segura et al., 2021).

Kriwet et al. (2009) erected the family Ptychotrygonidae to include sclerorhynchoid genera based on various unique features, casting several doubts on the presence of rostral denticles within Ptychotrygonidae. The absence of rostral denticles subsequently was confirmed by a complete skeleton of *Ptychotrygon rostrispatula* from the Turonian of Morocco that exhibits a rostrum without lateral rostral denticles (Villalobos-Segura et al., 2019a). Villalobos-Segura et al. (2019b) also assigned a second complete sclerorhynchoid skeleton from the Turonian of Morocco identified as *Asflapristis cristadentis* to Ptychotrygonidae.

Sclerorhynchoids display a wide paleogeographic distribution during the Cretaceous with most records coming from the northern hemisphere, such as North America, Europe, North Africa but also the Middle East (Cappetta, 2012). Conversely, sclerorhynchoid occurrences in the southern hemisphere are very rare, with some records from Oceania such as *Australopristis* from the Upper Cretaceous of New Zealand (Keyes, 1977; Martill & Ibrahim, 2012), or from South America including *Biropristis* from the Maastrichtian of Chile (Suárez & Cappetta, 2004), *Ischyrhiza* from the Maastrichtian of Argentina (Arratia & Cione, 1996) and Chile (Otero et al., 2013), *Pucapristis* from the Maastrichtian of Argentina (Cione & Pereira, 1985) and Bolivia (Gayet et al., 2001), and *Atlanticopristis* from the Albian-Cenomanian of Brazil (Pereira & Medeiros, 2008). Ptychotrygonids were widely distributed by the Cenomanian demonstrated by occurrences in Europe (Jonet, 1981; Bernárdez, 2002 ; Cappetta, 2004; Vullo et al., 2005; Kriwet et al., 2009), North America (Cappetta & Case, 1999), and North Africa (Werner, 1989), but all of these records are from the northern hemisphere. The new species reported here, from southernmost Patagonia, is thus the first ptychotrygonid record from South America, increasing the genus diversity and highlighting a significant paleogeographic range extension.

## Locality and Stratigraphic Settings

**Figure 1:**
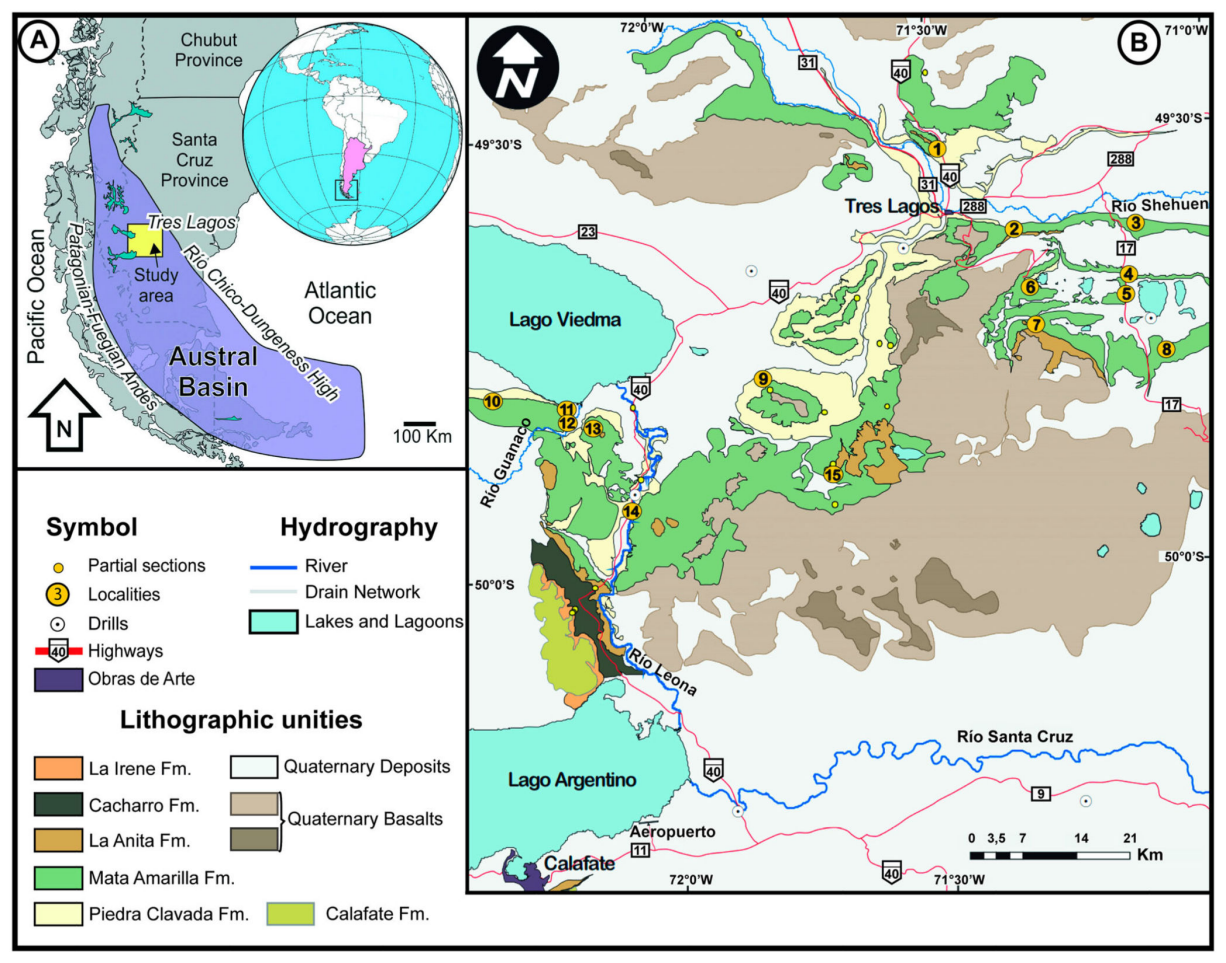
**A**, map of the Austral Basin and location of the study area; **B**, enlarged map of the Tres Lagos sector within the square shown in A. Locality No. 8 corresponds to 3LAG0 (modified from Varela, 2011; Varela et al., 2016).

**Figure 2:**
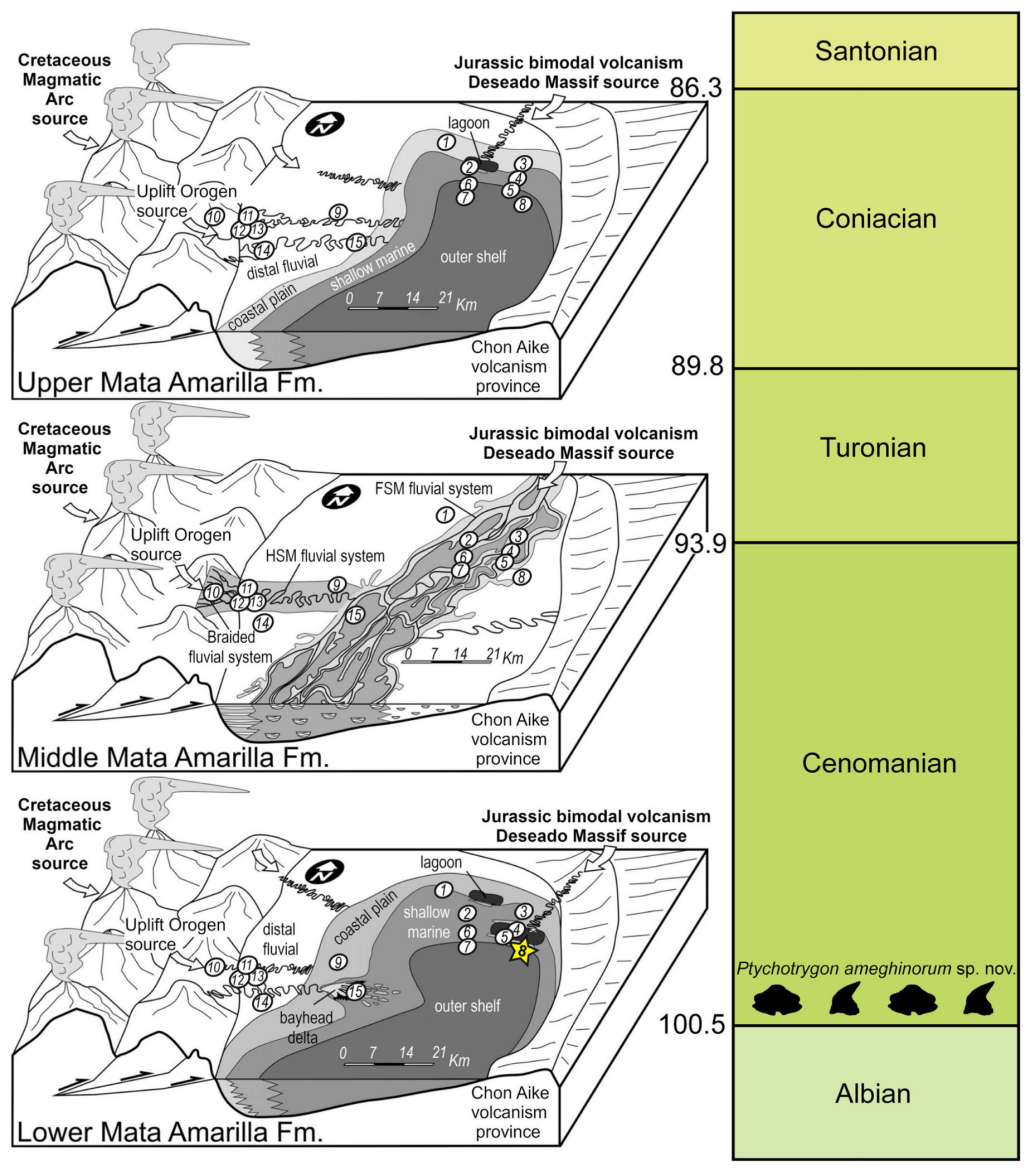
Changing landscape of the study area during the different phases of the sediment deposition of the Mata Amarilla Formation. Locality No. 8 corresponds to the locality 3LAG0 (from Varela et al., 2013).

Patagonia is the southern part of South America, including the southernmost area of Chile and Argentina. The southernmost sedimentary basin in South Patagonia is the Austral Basin (Fig. 1). It comprises the Rocas Verdes Marginal Basin that represents a back-arc basin with sediment infill ranging from the Late Jurassic to the Early Cretaceous, and the younger Magallanes Basin that corresponds to a foreland stage, ranging from the Early Cretaceous to the Cenozoic (Varela et al., 2012). The Austral Basin has been known to be rich in fossil remains since the 19th century (Ameghino, 1893), and many groups of Cretaceous vertebrates have been described from this basin. These include chondrichthyans (Bogan et al., 2016), dipnoans (Cione et al., 2007), sauropod and theropod dinosaurs (Novas et al., 2005, 2008), plesiosaurs (O’Gorman & Varela, 2010), and mammals (Rougier et al., 2011; Martin et al., 2022). The site where the new species described herein is located in the Santa Cruz Province of Argentina (Fig. 1). The fossiliferous deposits belong to the Mata Amarilla Formation (M.A. Fm.), which encompasses sediments ranging from the upper Albian to the lower Santonian (Varela, 2015, Varela et al., 2012). The sediments are divided in three parts: the first part corresponds to deposits of early Cenomanian to middle Cenomanian age, there is an uncertainty about the age of the basal part, which could be late Albian (Varela et al., 2012, 2018). The first part is characterized as an embayment with freshwater influx influenced by diverse deltaic and estuarine systems (Fig. 2). The eastern part is represented by sediments deposited in lagoons, bay-head deltas, estuaries and littoral marine paleoenvironments, whereas the western part is dominated by a distal-fluvial system (Varela, 2011). The middle part corresponds to deposits of middle Cenomanian to Turonian age and is characterized by a forced regression that led to the establishment of a fluvial system (Fig. 2). The upper part is of Coniacian age and it is also supposed that deposits from the top of that section extend into the lower Santonian (Varela, 2015; Varela et al., 2012). Transgressive deposits characterize this part of the M.A. Fm. that follow a second embayment of the Tres Lagos area.

The fossil remains of the new *Ptychotrygon* species derive from the lower section of the M.A. Fm, from site 3LAG0 (site 3LAG1 of Cione et al., 2007; Bajada de los Orientales of Varela et al., 2012). The locality is about 40 km east-south-east of the village of Tres Lagos (Fig. 1, No. 8). The fossil-bearing sediments represent the front of a deltaic-estuarine system, located at the frontier between the shallow marine and outer shelf environments (Fig. 2, BO).

## Material and Methods

The material described here was collected during an Argentinian–German field project funded by National Geographic Society and German Research Foundation (DFG) in 2009. At site 3LAG0 (see above), bulk sediments were collected from lower Cenomanian layers and subsequently screen-washed in the field. The concentrate was picked in the lab under a stereo microscope for vertebrate remains. Elasmobranch remains were subsequently sorted with the help of digital microscopes (Keyence VHX-1000 and VHX-6000). Some of the teeth were further processed and studied with a JEOL-JSM-6400 scanning electron microscope at the Department of Paleontology (University of Vienna, Austria) after having been sputter coated with gold (Sputter Coater SC 500). The systematic scheme and morphological terminology follow the one used by Kriwet et al. (2009), Cappetta (2012), and Villalobos-Segura et al. (2019a, b). All fossil material will be finally deposited in the Museo Regional Provincial Padre M. Jesús Molina, Río Gallegos, Santa Cruz Province, Argentina, with the prefix MPM-PV.

**Institutional Abbreviation**—**MPM-PV**, Museo Regional Provincial Padre M. Jesús Molina, Río Gallegos, Santa Cruz Province, Argentina.

## Systematic Paleontology

**Remarks**—We use Neoselachii sensu Compagno (1977) instead of Elasmobranchii sensu Maisey (2012) here, because we consider Elasmobranchii to also include non-neoselachian extinct shark-like chondrichthyans.

Class CHONDRICHTHYES Huxley, 1880

Sub-class ELASMOBRANCHII Bonaparte, 1838

Cohort EUSELACHII Hay, 1902

Sub-cohort NEOSELACHII Compagno, 1977

Super-order BATOMORPHII Cappetta, 1980

Order RAJIFORMES Berg, 1940

Sub-order SCLERORHYNCHOIDEI Cappetta, 1980

Family PTYCHOTRYGONIDAE Kriwet, Nunn, and Klug, 2009

**Included Genera**—*Ptychotrygon* Jaekel, 1894; *Ptychotrygonoides* Landemaine, 1991; *Texatrygon* Cappetta and Case, 1999; *Archingeayia* Vullo, Cappetta, and Neraudeau, 2007; *Asflapristis* Villalobos-Segura et al., 2019b.

Genus *PTYCHOTRYGON* Jaekel, 1894

**Type Species**—*Ptychodus triangularis* von Reuss, 1845.

**Diagnosis**—(emended from Villalobos-Segura et al., 2019a)— An extinct rajiform characterized by a hypertrophied rostrum lacking rostral denticles; two parallel, laterally located ventral rostral canals present; slender palatoquadrate and Meckel’s cartilage; second and third hypobranchials well-developed, close to each other and with no articulation surface with basibranchial; teeth small, less than 2 mm and oval-shaped, with a sharp, strong pyramidal crown and transverse crests; occasionally short transverse ridges present on labial crown face; labial apron variably developed and in some cases with a straight sagittal ridge on upper part; apron bent basally with a truncated projection; lingual crown face less developed than labial one; lingual uvula short and broad with central interlocking depression; labial face sigmoidal in profile view; root of holoaulacorhizous type with single pair of marginolingual foramina; root lobes sub-triangular in basal view; demarcation between the crown and the root is easily distinguished by a depression under the crown that forms a neck.

**Figure 3:**
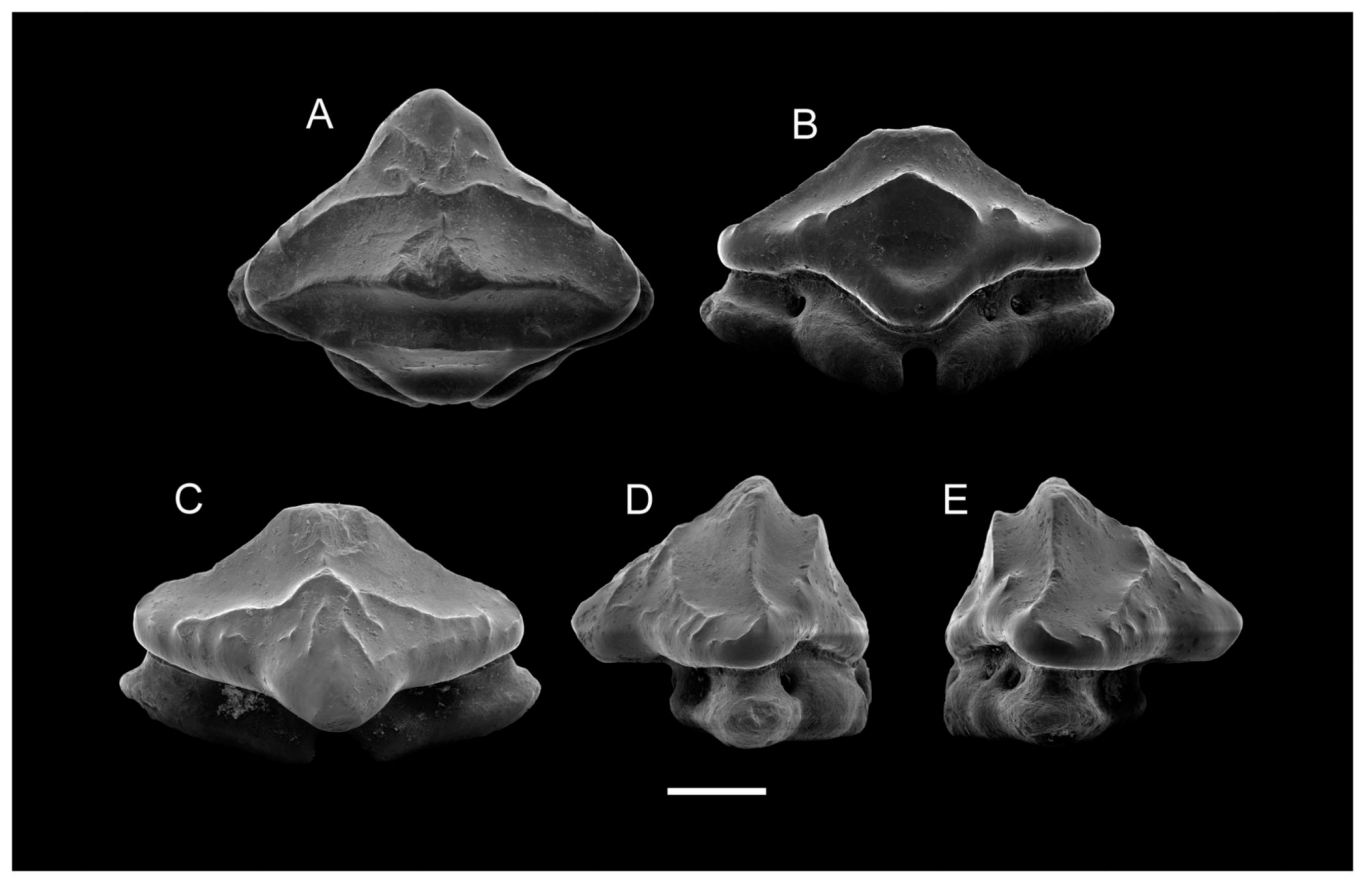
SEM images of the holotype of *Ptychotrygon ameghinorum* sp. nov., MPM-PV 1160.1.1, **A**, occlusal; **B**, lingual; **C**, labial; **D** and **E**, profile views. Scale bar equals 500 μm.

**Figure 4:**
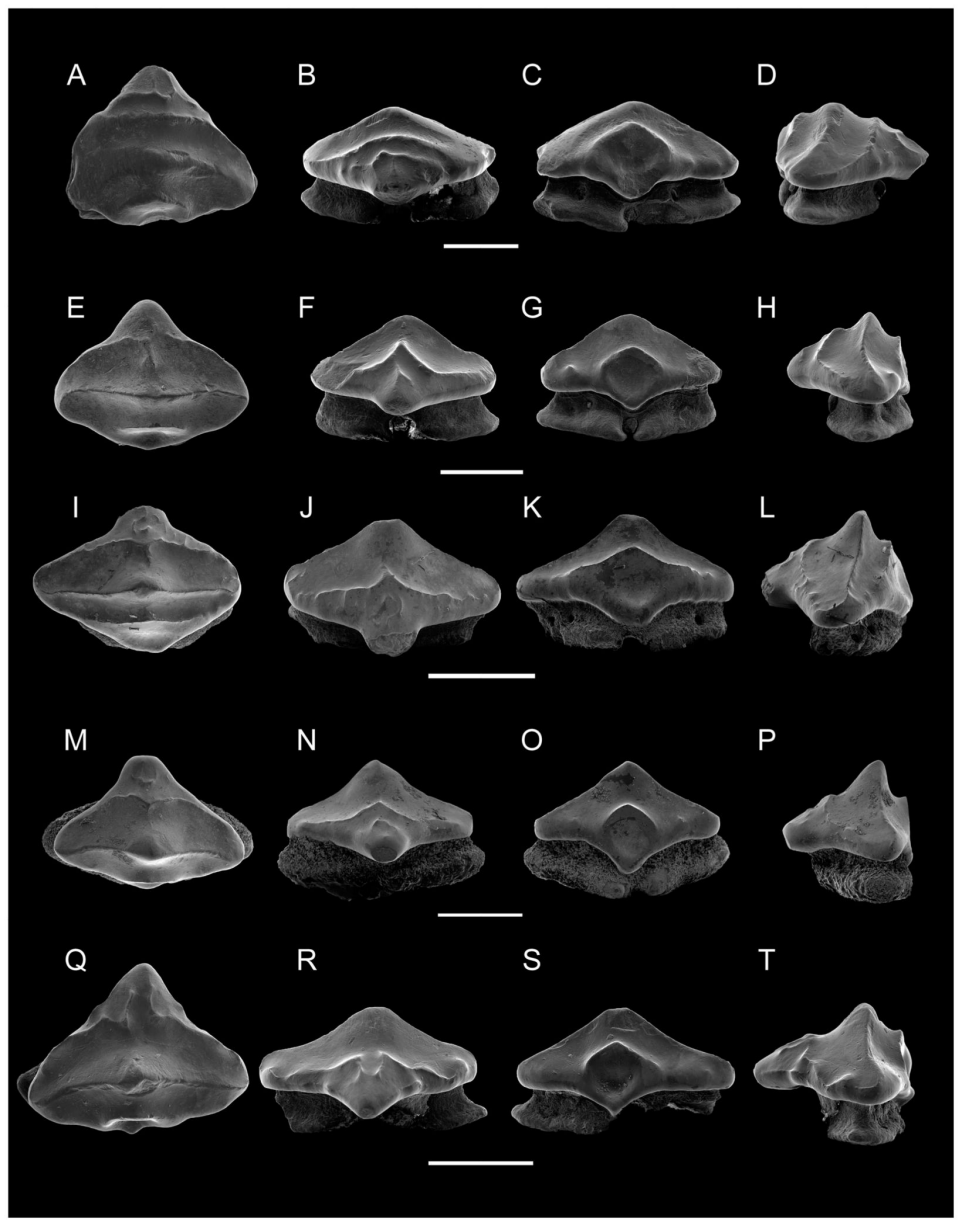
SEM images of paratypes of *Ptychotrygon ameghinorum* sp. nov., MPM-PV 1160.1.2, **A**, occlusal; **B**, labial; **C**, lingual; **D**, mesial views; MPM-PV 1160.1.3, **E**, occlusal; **F**, labial; **G**, lingual; **H**, mesial views; MPM-PV 1160.1.4, **I**, occlusal; **J**, labial; **K**, lingual; **L**, mesial views; MPM-PV 1160.1.5, **M**, occlusal; **N**, labial; **O**, lingual; **P**, mesial views; MPM-PV 1160.1.6, **Q**, occlusal; **R**, labial; **S**, lingual; **T**, mesial views. All scale bars equal 500 μm.

**Figure 5:**
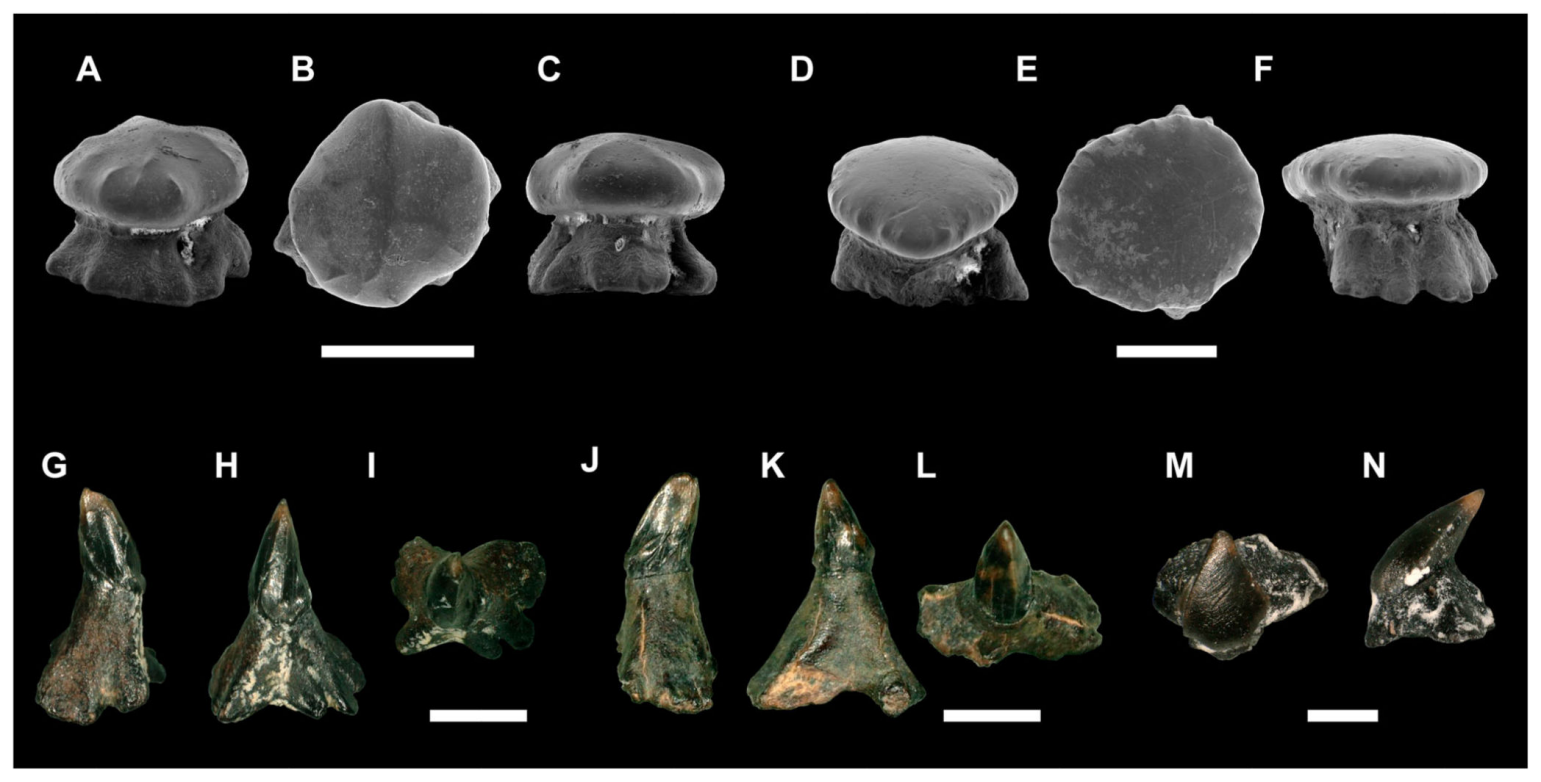
*Ptychotrygon ameghinorum* sp. nov. dermal denticles, MPM-PV 1160.1.42 (snout dermal denticles) **A**, profile; **B**, occlusal; **C**, profile views; MPM-PV 1160.1.43 (snout dermal denticles), **D**, profile; **E**, occlusal; **F**, profile views; MPM-PV 1160.1.44 (lateral cephalic denticles) **G**, profile; **H**, anterior; **I**, occlusal views; MPM-PV 1160.1.45 (lateral cephalic denticles) **J**, profile, **K**, anterior; **L**, occlusal views; MPM-PV 1160.1.46, **M**, occlusal; **N**, profile views. All scale bars equal 500 μm.

*PTYCHOTRYGON AMEGHINORUM* sp. nov. (Figs. 3–5)

**Holotype**—MPM-PV 1160.1.1. One anterior tooth.

**Etymology**—The species name is a tribute to the brothers Florentino and Carlos Ameghino, who were among the first Argentinian paleontologists to collect and study fossils from Patagonia during the 19th and early 20th centuries.

**Paratypes**—Forty teeth (MPM-PV 1160.1.2–41) representing anterior to lateral teeth.

**Additional Material**—Six dermal denticles (MPM-PV 1160.1.42–47).

**Type Locality**—3LAG0 (49°45′49.5′′S, 71°05′13.1′′W), 40 km ESE of the town of Tres Lagos, Santa Cruz Province, Patagonia, Argentina.

**Stratigraphic Age**—Lower part of the Mata Amarilla Formation, early Cenomanian, Upper Cretaceous.

**Diagnosis**—A species of *Ptychotrygon* characterized by three well-developed transverse crests on the oral tooth crown, the median transverse crest being the best-developed; a well-developed, sharp, and erected central cusp may be present; apron well-developed, rounded, bearing small ridges; lingual uvula short, broad with a rounded lingual extremity; a sharp and abrupt lingual transverse crest, two and an additional marginal foramina are present on lingual root face.

**Description**—The holotype (MPM-PV 1160.1.1) is an anterior tooth having three transversal crests on the crown, of which the median one is the best developed. This crest extends almost completely transversely across the crown, separating the labial and the lingual portion of the crown. It is continuous and forms a well-defined central cusp, but becomes less marked laterally and finally curves lingually before it connects to the labial crest (Fig. 3C). The labial crest, conversely, is less developed but extends to the distal and mesial crown edges, separating the apron from the rest of the crown (Fig. 3A, C). Additional short, vertical ridges occur margino-labially on each side of the apron, accompanied by some short, undulating horizontal ones below the labial crest.

The lingual crest forms the apical border of the lingual depression and divides the lingual face in a narrow upper portion, which is concave in lateral views, and a steep lower portion that continues into the lingual uvula. Although the lingual crest is less marked and shorter than the medial crest and does not reach the margins of the crown, it is better developed than the labial one. The lingual uvula is short and broad, with a rounded linguo-basal extremity.

The root is significantly lower than the crown and with two wide labial lobes seen in labial view. Each lobe is triangular in basal view, with smooth and rounded angles. The basal surfaces of the root lobes are flat and oblique in labial and lingual views. The central nutritive foramen is narrow. There are two and one margino-lingual foramina, respectively.

Paratype MPM-PV 1160.1.2 (Fig. 4A–D), is an incompletely preserved anterior tooth, with part of the distal crown edge missing. In occlusal view, the tooth is triangular with steep and only medially slightly convex margino-labial edges. It is characterized by short transverse ridges associated with vertical ridges on the apron in addition to the three characteristic transverse crests of this species (Fig. 4A). The vertical ridges are faint and continue to the apex of the apron. Three transverse crests are present on the oral face with the medial crest being less developed than the medial crest in the holotype (Figs. 3D, E, 4D). Additionally, the central cusp is small, wide and has a rounded angle. The labial ridge is well-developed and bears irregular occlusal crests, as does the ridge on the apron. The crown shape is triangular in labial and lingual views (Fig. 4B, C), and the apron is wide mesio-distally and short. The uvula also is considerably wide. The lingual face is steep and short with a pronounced medial depression for tooth interlocking. This depression is roofed by a prominent, concave ridge, which follows the occlusal outline of the transversal crest in lingual view. The root is low and slightly wider than the crown. The narrow part of the right root lobe close to the furrow is not preserved, and the distal edge extremity of the left root lobe is also missing (Fig. 4C). A single pair of foramina is present on the root rather than one pair and a single one on the holotype. The right lobe close to the furrow is only partly preserved, as is the left root lobe’s distal edge extremity (Fig. 4C). A single pair of marginolingual foramina is present on the root, differing from the condition in the holotype.

Paratype MPM-PV 1160.1.3 (Fig. 4E–H), also represents an anterior tooth and it displays the typical three transverse crests on the oral face. The apron is rather accentuated and well set off from the tooth crown in occlusal view. There is no transversal ridge developed on the apron. The labial crest is rather faint and tectiform in labial view, and a short and vertical sigmoidal ridge starts at the labial transverse crest and extends apically towards the medial one, is present on the labial crown face (Fig. 4E). In occlusal view, the margino-labial edges are convex at the transition from the crown to the apron. The labial transverse crest is interrupted centrally, whereas the medial crest is continuous and rises centrally into a well-developed wide central cusp with a rounded apex (Fig. 4E–G). Laterally, the medial crest curves lingually. The lingual face exhibits a large and rounded central depression, which is roofed by a short, convex but well-marked crest. The uvula is short and narrow, overhanging the root only slightly. A pair of margino-lingual foramina (plus an additional one) is present on the left root lobe, instead of on the right lobe as in the holotype.

Paratype MPM-PV 1160.1.4 (Fig. 4I–L) is an asymmetrical antero-lateral tooth, with a better developed distal than mesial crown shoulder. The margino-labial edges are rather straight but convex at the transition to the apron. The apron is well set off from the crown but short and blunt in occlusal view. There are some vermiculate, generally vertically oriented ridges on the apron and the distal margino-labial face below the labial crest. Three transverse crests are present. The medial transverse crest is rectilinear across the complete crown length, before it curves and reaches the labial crest at the margino-labial edges. Centrally, a well-developed central cusp on the median crest is developed, the apex of which is slightly abraded. The lingual ridge is broadly arched concavely and roofs a shallow lingual depression for tooth interlocking. Laterally, the lingual crest continues marginally and becomes irregular at the lateral extremities of the crown’s lingual face. The uvula is short, accentuated, and overhangs the root. The root is not well preserved and shows traces of abrasion. Nevertheless, a pair of marginolingual foramina is preserved, with an additional mesial one.

Paratype MPM-PV 1160.1.5 (Fig. 4M–P) most likely represents an anterolateral tooth and is characterized by a triangular tooth crown with slightly lingually curved lateral crown edges. The margino-labial edges are straight and convex, respectively, and continuous with the apron. The apron is short, wide, and blunt. There is a short, transverse and irregular ridge developed on the apron. The labial ridge is rather weak and medially arched towards the central cusp in occlusal view, whereas the lateral portions are curved, reaching the lateral edges of the crown. A faint vertical ridge ascends from the labial ridge centrally, where it is incised extending apically a short distance, but not reaching the apex. This vertical ridge also is present in paratypes MPM-PV 1160.1.3 and MPM-PV 1160.1.4, but not in MPM-PV 1160.1.2 and MPM-PV 1160.1.6. The medial crest is continuous mesio-distally across the tooth crown, reaching the lateral edges of the crown shoulders. Laterally, the crest is curved lingually, centrally it forms a well-demarcated and acute central apex. The lingual face is abrupt and steep. The lingual crest is short and arched apically, roofing the lingual depression, slightly oval in outline, continuing onto the uvula. The uvula is short, narrow, accentuated, and overhangs the root. The root bears two massive and broad root lobes, which are separated by a narrow and shallow nutritive groove. It is not possible to establish the number of marginolingual foramina because the root seems to be slightly re-crystallized or covered by minute particles. In occlusal view, it juts out labio-laterally below the crown.

Paratype MPM-PV 1160.1.6 (Fig. 4Q–T) appears to represent a lateral tooth, as is quite distinctive from all other paratypes in that it is triangular in occlusal view with an elongated and acute apron. Nevertheless, the tooth is elongated mesio-distally in labial and lingual views, supporting its assignment to a lateral tooth row.

The labial crest is irregular and incomplete centrally, where the two mesial and distal portions are recurved anteriorly and extend onto the apron. Additional short, almost knob-like vertical ridges are present margino-labially (Fig. 4R), similar to the condition observed on the holotype (Fig. 3C). The medial crest is continuous across the tooth crown and rises centrally into a triangular apex. In labial and lingual views, the crown is low and mesio-distally elongated with the distal crown being lower and more elongated than the mesial one. The lingual crest is broadly arched apically, roofing a shallow and basally far-reaching central depression. The lingual face is steep and reduced as on all other specimens. The apex is somewhat damaged lingually. A small tubercle is visible lingually on the mesial crown shoulder that resembles the condition seen in MPM-PV 1160.1.4 (Fig. 4O). This tubercle seems to be a remnant of the lingual crest that is slightly extended distally in MPM-PV 1160.1.5 (Fig. 4S) and only separated from the lingual crest in MPM-PV 1160.1.4 (Fig. 4K) by a small gap. The root is incomplete, with most of the distal lobe missing. The distal lobe is better preserved and displays a pair of margino-lingual foramina. The basal face of this root lobe is almost horizontal.

**Dental Variation**—Small dental variations can be observed in *P. ameghinorum* sp. nov. Accordingly, most of the teeth possess a well-developed central cusp and an additional marginal foramen, which is missing, in other teeth (e.g., MPM-PV 1160.1.2; Fig. 4A–D). The position of the additional lingual foramen varies and either is located on the right (distal?) lobe in the holotype or on the distal lobe in several paratypes (Fig. 4G, K). The marginal foramen position inversion could indicate a symmetrical pattern in the jaw rami.

The occlusal ornamentation varies to some degree labially with respect to the apron and/or labial crest, which might be related either to position in the jaws or could indicate some sort of sexual dimorphism. This, however, can only be established with more articulated skeletal material and thus remains very hypothetical. We nevertheless do not deem the differences to be sufficient to assign the teeth to two different species. The presence of an additional, fourth ridge on the apron resembles the situation seen in the Campanian species *P. boothi*. However, this ridge never is as well developed as the three crests, which is a common morphological feature of the new species. The apron length and form also vary between specimens and can be quite short or well extended labially (Fig. 4D, T). Additionally, the apex ranges from being low and broad to high and accentuated, which we consider to represent variations within the dentition. Thus, the dental variation indicates the dentition to be gradient monognathic.

**Dermal Denticles**—Two types of dermal denticles were recovered from site 3LAG0 in association with teeth of *P. ameghinorum* sp. nov. The first morphotype possesses a rounded, globular crown with small vertical folds along the lateral margins. The apical part of the crown is relatively flat, mostly smooth, and irregular in outline in apical view. However, a weak transverse and lightly pronounced crest was observed apically in one denticle (Fig. 5B). The root is wide at its base and decreases in size apically forming a narrow collar between root and crown. Foramina occur in the upper part of the root, below the neck (Fig. 5C, F). By their rounded and relatively smooth aspect, these denticles might come from the snout region, which is similar to what is found in many neoselachians (Thies & Liedner, 2011).

The second morphotype resembles rostral denticles to some extent, as they are characterized by a high root and a shorter, slightly posteriorly inclined cusp (Fig. 5G–L). The cusp is laterally compressed, devoid of any distinct anterior and posterior cutting edges, but displays faint ridges on its lateral sides. The root is asymmetrical in anterior and posterior views with a flaring base that is basally divided by a shallow and broad furrow (Fig. 5 I, J). In apical view, the base is well extended laterally below the cusp, but less so antero-posteriorly. In lateral view, the root is high and antero-posteriorly narrow. The morphology of this denticle corresponds to those found commonly in the lateral cephalic region of sclerorhynchoids, which can be clearly distinct from sclerorhynchoid rostral denticles which are enlarged and associated with a well-developed cusp (Wueringer et al., 2009; Welten et al., 2015). Another type of lateral cephalic denticle also was found in 3LAG0 deposits, differing from the first one described above by a shorter root and reduced denticle height (Fig. 5M, N). The crown also is more compressed antero-posteriorly forming blunt lateral cutting edges. This second type of cephalic denticle, by its reduced height, was positioned more distally to the rostrum than the elongated cephalic denticles described above.

**Taxonomic Comparison**—The new taxon can be clearly identified as a species of *Ptychotrygon* based on a combination of morphological features that are different to other ptychotrygonid genera:

- *Archingeayia* Vullo, Cappetta, and Néraudeau (2007), described from Cenomanian deposits of France, differs from *Ptychotrygon* teeth by having a more reduced labial crown face, labio-lingually compressed teeth, absence of apron, a single occlusal crest, and the absence of a lingual depression in most teeth (where it is present, there is no lingual crest roofing the depression).
- *Asflapristis* Villalobos-Segura, Underwood, Ward, and Claeson (2019b) based on skeletal remains from the Turonian of Morocco, differs from *Ptychotrygon* by homodont teeth that are wider than high, rounded, with a weak labial apron, lack a central cusp, and a well-pronounced oral ornamentation consisting of small and connected ridges.
- *Ptychotrygonoides* Landemaine (1991), described from the Cenomanian–Turonian of Europe, is characterized by teeth that are much broader antero-posteriorly than labio-lingually, a less marked uvula with a well-marked depression, a very abrupt lingual face, a well-developed basal bulge of the root, a strongly cuspidate crown, an extended labial crown face, and a wide but stout apron. These characters, however, occur variously in species of *Ptychotrygon*, which is why Kriwet et al. (2009) considered *Ptychotrygonoides* to be synonymous with *Ptychotrygon*.
- *Texatrygon* Cappetta and Case (1999), described from the Late Cretaceous of North America, can be distinguished from *Ptychotrygon* by a high and triangular tooth crown, the absence of transversal crests, a very different ornamentation pattern on the labial side of the crown and the presence of a pustule above the lingual depression instead of a crest.

Twenty-two species of *Ptychotrygon* have been described thus far, and theses differ from *Ptychotrygon ameghinorum* sp. nov. by the presence or absence of diverse characteristics and by chronostratigraphic and spatial ranges:

- *P. agujaensis* McNulty and Slaughter (1972), described from Campanian deposits of North America, can be differentiated from P. ameghinorum sp. nov. by having lower teeth with only one poorly developed transverse crest.
- *P. blainensis* Case (1978), described from Campanian deposits of North America, differs from P. ameghinorum sp. nov by teeth having reduced labial and lingual crests, a characteristic labial ornamentation extending to the central cusp, a higher and more triangular crown, and a single pair of marginal foramina.
- *P. benningensis* Case, Schwimmer, Borodin, and Leggett (2001), described from Santonian deposits of North America, can be differentiated from *P. ameghinorum* sp. nov. by higher and more massive teeth that are less extended margino-distally, a reduced labial crest, and a shorter and wider apron.
- *P. boothi* Case (1987), described in Campanian deposits of North America, has teeth that resemble those of *P. ameghinorum* sp. nov., but differs in the presence of four transverse crests, and a less pronounced uvula below the lingual depression.
- *P. chattahoocheensis* Case, Schwimmer, Borodin, and Leggett (2001), described from Santonian deposits of North America, differs from *P. ameghinorum* sp. nov. by having poorly pronounced transverse crests, associated with a very weak ornamentation, and a more extended uvula overhanging the root on the lingual face, where only a single pair of marginal foramina is present.
- *P. clementsi*, Case, Cook, Sadorf, and Shannon (2017), described from upper Maastrichtian deposits of North America, differs from *P. ameghinorum* sp. nov. in having weak or even lacking transverse crests, the absence of any ornamentation, and lack of an additional marginolingual foramen.
- *P. cuspidata* Cappetta and Case (1975a), described from lower Maastrichtian deposits of North America, differs from *P. ameghinorum* sp. nov. by having a massive, rounded, and high main cusp, the absence of transverse crests.
- *P. ellae* Case (1987), described from Campanian deposits of North America, resembles *P. ameghinorum* sp. nov. to some degree in having three transverse crests with the median one being as pronounced as the anterior of *P. ameghinorum* sp. nov. It nevertheless can be easily distinguished from the new species from Patagonia in having an even more reduced lingual face, a more rounded lingual depression, and the presence of additional lateral foramina on each side of the root.
- *P. eutawensis* Case, Schwimmer, Borodin, and Leggett (2001), reported from Santonian deposits of North America, differs from *P. ameghinorum* sp. nov. by lacking labial and lingual transverse crests, absence of a central cusp, and a reduced crown height.
- *P. greybullensis* Case (1987), reported from Campanian deposits of North America, differs from *P. ameghinorum* sp. nov. in lacking a labial transverse crest, lacking an additional foramen, and lacking of any ornamentation on the labial side of the crown.
- *P. geyeri* Kriwet (1999b), reported from the late Albian to the early Cenomanian of Spain, differs from *P. ameghinorum* sp. nov. in having a less pronounced lingual face and a less developed lingual depression.
- *P. gueveli* Cappetta (2004), reported from Turonian deposits of France, can be differentiated from *P. ameghinorum* sp. nov. by a well-developed ornamentation consisting of irregular and anastomosing ridges, the presence of only one transverse crest which is less marked than in the new species described here, and a more massive bulbous crown.
- *P. henkeli* Werner (1989), described from the Cenomanian of Egypt, resembles *P. ameghinorum* sp. nov. in having a strong lingual crest and a well-marked median crest. It, nevertheless, can be differentiated based on the presence of ridges covering the median part of the crown, the presence of two pairs of marginolingual foramina, and a wider lingual depression.
- *P. ledouxi* Cappetta (1973), reported from Turonian deposits of North America, differs from *P. ameghinorum* sp. nov. in having lower teeth, a less marked median transverse crest, and more compact anterior teeth.
- *P. pustulata* Kriwet, Nunn, and Klug (2009), described from upper Albian to lower Cenomanian deposits of Spain, can be distinguished from *P. ameghinorum* sp. nov. by less marked transverse crests, a central cusp that is not well differentiated and a significantly shorter apron.
- *P. rostrispatula* Villalobos-Segura, Underwood, and Ward (2019a) based on complete skeletal remains in association with articulated dentitions, from Santonian deposits of Morocco, resembles *P. ameghinorum* sp. nov. in their tooth morphology. However, the Moroccan species can be differentiated from the new species by a less marked and shorter apron, associated with reverse V-shaped labial crest centrally forming a chevron-like structure, a less marked central cusp, and by a root with a convex basal face.
- *P. rugosum* Case, Schwimmer, Borodin, and Leggett (2001), described from Santonian deposits of North America, differs from *P. ameghinorum* sp. nov. in having teeth with a relatively flat occlusal surface associated with reduced transverse crests, a bulky root, and a shorter median face associated with a wide and square apron.
- *P. slaughteri* Cappetta and Case (1975b), reported from Cenomanian deposits of North America, exhibits a dental morphology similar to that of *P. ameghinorum* sp. nov. in occlusal view. Nevertheless the Texan species can be distinguished from the Patagonian species by an oblique labial face, a median crest that extends lingually, and a more marked lingual depression in association with a less developed lingual transverse crest.
- *P. striata* Kriwet, Nunn, and Klug (2009), described from the lower Cenomanian of Spain, can be differentiated from *P. ameghinorum* sp. nov. by the higher and wider tooth crown and central crest and the presence of well-marked vertical folds on the lingual and labial sides around the central cusp.
- *P. triangularis* Reuss (1845), reported from the Cenomanian to the Coniacian of Europe and North America, differs from *P. ameghinorum* sp. nov. in having a strong ornamentation on the occlusal crown surface that is relatively flat and lacks a well-marked median cusp.
- *P. vermiculata* Cappetta (1975), described from lower Maastrichtian deposits of North America, resembles *P. ameghinorum* sp. nov. in the shape of the root in basal view and the presence of an additional foramen. However, the median transversal crest is more developed and associated with a central cusp in *P. ameghinorum* sp. nov., while in *P. vermiculata* the transverse medial crest is wide and rounded, the labial face bears a vermiculate ornamentation. The crown is also comparably higher in the new species from Patagonia.
- *P. winni* Case and Cappetta (1997, reported from the Maastrichtian of North America, differs from *P. ameghinorum* sp. nov. in having teeth with a more reduced lingual face, a deeper and wider root, a poorly developed labial ridge, and a prominent cusp. An ornamentation of sigmoidal ridges is also present on the occlusal surface, which can form more or less parallel lines on the labial face of the crown.

## Discussion

### Evolutionary Considerations

Members of Ptychotrygonidae can be easily distinguished from all other sclerorhynchoids by the absence of lateral and ventral rostral denticles, as revealed by skeletal remains of *Ptychotrygon* and *Asflapristis*. It therefore can be hypothesized that rostral denticles were lost once in the evolution of sclerorhynchoids and their absence is a synapomorphy of ptychotrygonids, rather than a plesiomorphic trait for the total clade Sclerorhynchoidei. The oldest teeth and rostral denticles of sclerorhynchoids were reported from the Barremian and Aptian (Early Cretaceous), respectively, and can be assigned to non-ptychotrygonid sclerorhynchoids (Thurmond, 1971; Kriwet, 1999a), while the oldest records of ptychotrygonids come from the Albian (Kriwet, 1999b). This temporal distribution is in accordance with the assumption that rostral denticles were lost within sclerohynchoids and represents an apomorphic rather than a plesiomorphic trait, bearing in mind that the fossil record, of course, is highly biased.

### Taxonomic Implications

**Figure 6:**
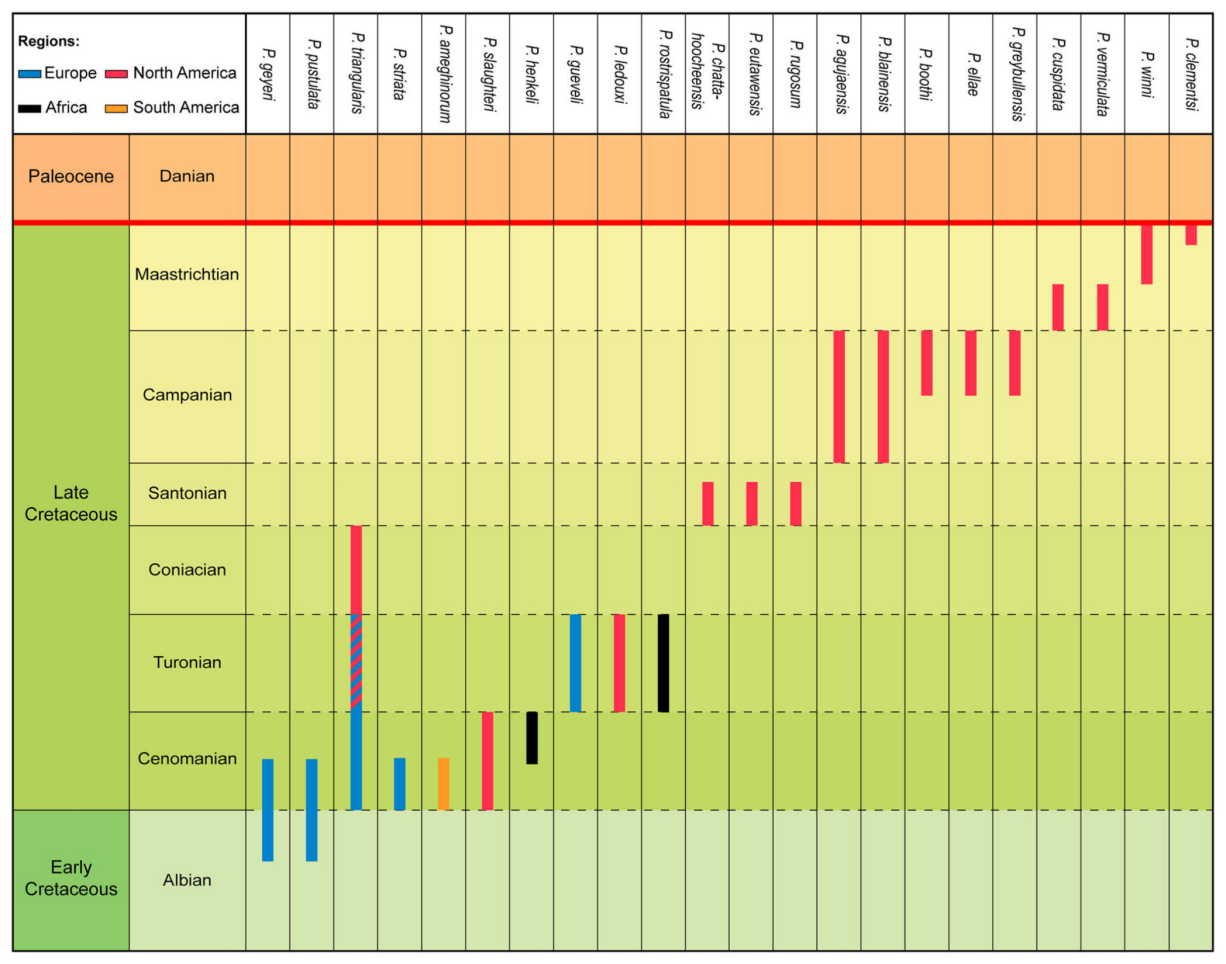
Chronostratigraphic range of the 22 *Ptychotrygon* species across the Cretaceous record.

The stratigraphic ranges of the 22 described *Ptychotrygon* species from the Cretaceous highlight several peculiarities (Fig. 6). Several species seemingly have the same spatial and temporal range, such as *P. benningensis*, *P. chattahoocheensis*, *P. eutawensis*, and *P. rugosum*, all from the lower to middle Santonian of North America (Case et al., 2001), or *P. boothi, P. ellae*, and *P. greybullensis* from upper Campanian deposits of North America (Case, 1987). Both, *P. boothi* and *P. ellae* (Case, 1987) occur in the same locality and formation (Case Estuary, Mesaverde Fm., Wyoming) and display very similar morphologies. The main difference is the presence of a well-developed central cusp on the medial transverse crest in the tooth crowns of *P. boothi*, which is absent with *P. ellae* and suggests a case of sexual dental dimorphism (see below). We suggest, following Article 23 of the International Code of Zoological Nomenclature, to consider these two Campanian species as synonyms and, by applying the priority principle of the ICZN article 23.1, to synonymize *P. ellae* with *P. boothi*.

The two Santonian species, *P. benningensis* and *P. rugosum*, described by Case et al. (2001) from the Eutaw Formation (Georgia, U.S.A.) were discussed by Cicimmuri et al. (2014) and *P. benningensis*, described first as *Erguitaia benningensis*, consequently was assigned to *Texatrygon*, while the species *rugosum*, described first as *Erguitaia rugosa* by Case et al. (2001) and then attributed to *Ptychotrygon* by Cappetta (2006), was retained as *P. rugosum*. The attribution of the species *P. rugosum* to the genus *Ptychotrygon* is doubtful considering its tooth morphology. This species is characterized by a hexagonal tooth crown in occlusal view and an occlusal surface that is characterized by numerous but short transverse crests that are mainly located on the labial occlusal face. The occlusal face is relatively flat and continues into a broad rectangular apron. The lingual crown ornamentation is characterized by anastomosing and discontinuous ridges, which are associated with a shallow central depression on the lingual face. This tooth morphology is more reminiscent of teeth of *Asflapristis cristadentis*, described from the Turonian of Morocco (Villalobos-Segura et al., 2019b) than of that of *Ptychotrygon*. The species *Asflapristis rugosa* comb. nov. (Article 34.2 and 48 of the ICZN) differs from *A. cristadentis* in a deeper tooth root, more transverse crests and an ornamentation that is restricted to the lingual face instead of to the labial face of the tooth crown as in *A. cristadentis*. The new combination strongly increases the spatial distribution and stratigraphic range of the genus *Asflapristis* that was only described from the Turonian of North Africa, and now is also known from the Santonian of North America.

Important characters are not described for *P. eutawensis* (Case et al., 2001). For further comparison and diagnosis, holo- and paratypes should be re-investigated.

The species *P. clementsi* was erected by Case et al. (2017) based on two isolated teeth, corresponding to the holotype and the paratype. These specimens lack well-marked transverse crests and occlusal or margino-labial ornamentations, and are rather globular and blunt, due to the poor preservation state. Consequently, the assignment of these specimens to a distinct species of *Ptychotrygon* is rather ambiguous and we suggest considering them as *Ptychotrygon*? sp. Therefore, *P. clementsi* should be regarded as a nomen dubium.

### Sexual Dental Dimorphism

The absence of a well-developed cusp on the transverse medial crest in several teeth of the new species described herein probably indicates sexual dental dimorphism. Sexual dimorphic patterns are well known in the dentition of various extant and extinct batomorphs and selachiomorphs (e.g., Cappetta, 1973; Kajiura & Tricas, 1996; Underwood et al., 1999; Everhart, 2007; de Sousa Rangel et al., 2016; Berio et al., 2020). Especially in batomorphs, males display at least seasonally different tooth morphologies from females, in that their teeth develop an accentuated cusp, which is used to hold on to the pectoral fins of the female during copulation. Sexual dental dimorphic patterns in ptychotrygonids already were suggested by Case et al. (2001). We consequently assume, even though the sample of teeth of the new species described here is limited, that this species had sexually dimorphic dental patterns.

### Dermal Denticles

The dermal denticles recovered from site 3LAG0 representing two distinct morphotypes correspond well to those known from the skeletal remains of *Asflapristis* (Villalobos-Segura et al., 2019b). In this respect, the first morphotype (Fig. 5A–F) resembles rounded dermal denticles in the mouth region of *Asflapristis*. Incomplete dermal denticles found in association with *A. cristadentis* and described as thorn-like rostral denticles (Villalobos-Segura et al., 2019b), seem to correspond to the second type of dermal denticles described above (Fig. 5G–N). Similar denticles are reported in association with ptychotrygonid teeth from the Late Cretaceous of France and England, described as sclerorhynchoid indeterminate and *Micropristis*? sp. rostral teeth (Vullo et al., 2003; Guinot et al., 2012). These dermal denticles, like those of *Sclerorhynchus atavus*, belong to the antero-lateral cephalic region (Wueringer et al., 2009, Welten et al., 2015). Similar fossils of *Kiestus texana* and *Ischyrhiza mira*, identified firstly as rostral denticles, possess a similar morphology to the second type of dermal denticles described here (Jansen et al., 2012; Hoganson et al., 2019). However, a preserved rostrum of *Ischyrhiza mira* shows that the rostral denticles of *Ischyrhiza* are very different to those of morphotype 2 described here (Sternes & Shimada 2019

This also implies that the elements identified as rostral denticles by Jansen et al. (2012) and Hoganson et al. (2019) are in fact dermal denticles from the antero-lateral cephalic region. Confusion of cephalic dermal denticles and rostral denticles results from similar morphologies of these structures in different sclerorhynchoid genera, such as *Micropristis* or *Sclerorhynchus* (Cappetta, 1981; Vullo et al., 2003; Guinot et al., 2012; Welten et al., 2015). The development of cephalic dermal denticles and rostral denticles, even though superficially similar, correspond to a regionalization of the modified rostral and cephalic denticles as hypothesized by Welten et al. (2015).

*Ptychotrygon ameghinorum* sp. nov. is the only attested sclerorhynchoid taxon based on the isolated teeth from 3LAG0 deposits that are mainly dominated by dipnoans and teleosteans. Instead of teeth, lateral cephalic denticles of sclerorhynchoid do not bear significant information supporting a diagnosis. Besides, the cephalic denticles described here cannot be attributed to *Kiestus*, *Ischyrhiza*, or any sclerorhynchoid by the absence of teeth related to these taxa in 3LAG0 deposits. By the presence of *Ptychotrygon ameghinorum* sp. nov. in association with lateral cephalic denticles in 3LAG0 deposits, and the absence of additional sclerorhynchoid taxa in 3LAG0 layers, the attribution of these lateral cephalic denticles to *Ptychotrygon ameghinorum* sp. nov. is the parsimonious decision.

### Stratigraphy and Paleobiogeography

*Ptychotrygon ameghinorum* sp. nov. is the first reported and described species of *Ptychotrygon* from the southern hemisphere. Previously scarce material from late Campanian to Maastrichtian Peruvian deposits was assigned to *Ptychotrygon* but without species attribution (Cappetta in Jaillard et al., 1993). However, the preservation of the single tooth is poor, as only a part of the crown is preserved and the authors provided no illustration. This material occurrence has not been referred at a later date by the author in Cappetta (2004, 2012). Therefore, we consider this record as doubtful.

The oldest records of *Ptychotrygon* are from the Albian (*P. geyeri* Kriwet, 1999b) and Albian to lower Cenomanian (*P. pustulata* Kriwet, Nunn, and Klug, 2009) from Spain. This suggests that the center of origin for this genus was somewhere in southwest Europe, probably on the Iberian Peninsula. However, this needs to be corroborated by additional diversity analyses, which are not the focus of this study. *Ptychotrygon* displayed its greatest taxonomic diversity and distribution during the Cenomanian, with the first records outside Europe in North Africa, South and North America (Fig. 6). The Coniacian saw the lowest diversity, *P. triangularis* (Reuss, 1845) being the singular species reported from this time interval in North America (Hamm & Cicimurri, 2011). This low diversity, however, most likely represents a collecting bias, because the diversity of *Ptychotrygon* before (Turonian) and after (Santonian) the Coniacian was significantly larger. The stratigraphic and geographic distribution of *Ptychotrygon* apparently increased significantly from the Santonian to the Maastrichtian but all described *Ptychotrygon* species are from North America. This seemingly diverging occurrence pattern in North America and the rest of the world could represent also a collecting bias rather than a real diversity signal and indicates that the North American fossil record is obviously better sampled whereas European marine deposits apparently were less studied. Although *Ptychotrygon* was reported from the Maastrichtian of Morocco and Egypt, these specimens were not identified at the species level (Cappetta, 1991; Noubhani & Cappetta, 1997). Our short stratigraphic and paleobiogeographic review demonstrates that our knowledge of *Ptychotrygon* in terms of species abundances and occurrences still is very incomplete. But it is obvious that *Ptychotrygon* was a victim of the K/Pg boundary event (see also Kriwet & Benton, 2004).

## Conclusion

**Figure 7:**
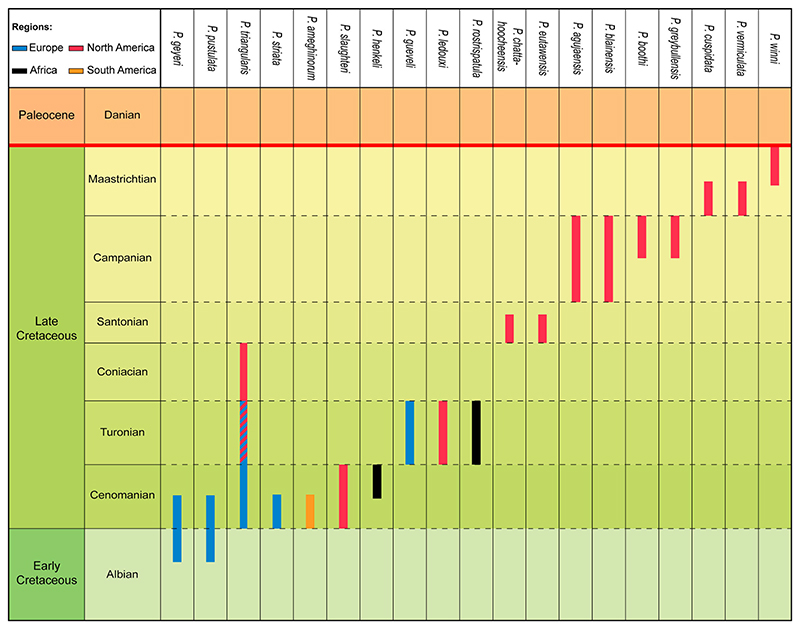
Re-evaluation of the chronostratigraphic range of the 19 *Ptychotrygon* species across the Cretaceous record.

*Ptychotrygon ameghinorum* sp. nov. is the first unambiguous species of *Ptychotrygon* from the southern hemisphere and represents the southern-most record of ptychotrygonids, increasing its known distribution significantly. A review of all 22 previously described species demonstrated that some taxa have to be either synonymized or transferred to other ptychotrygonid genera. This leaves 19 valid species including *P. ameghinorum* sp. nov. (Fig. 7), with a stratigraphic range from the Albian to the Maastrichtian. During the early Late Cretaceous (Cenomanian–Turonian), *Ptychotrygon* is well known and distributed in the Tethyan realm in North Africa, Europe, and North America (Werner, 1989; Cappetta & Case, 1999; Cappetta, 2004; Vullo et al., 2005; Kriwet et al., 2009; Néraudeau et al., 2016). The first occurrence of *Ptychotrygon* in the Cenomanian of the Santa Cruz Province in southern Patagonia is striking, because southern Patagonian elasmobranch associations, at least in the Maastrichtian, are dominated by cold-water taxa with affinities to the Weddellian Bioprovince such as *Paraorthacodus* or *Notidanodon* (Ameghino, 1893; Bogan et al., 2016). Northern and central Patagonian Maastrichtian elasmobranch faunas, conversely, show strong Tethyan influences during the Late Cretaceous, including warm-water taxa such as as *Serratolamna, Squalicorax*, or *Hypolophodon* (Bogan & Agnolin, 2010; Bogan & Gallina, 2011). The faunal differentiations in Patagonia thus seem to be strongly influenced by ocean circulation patterns. Sclerorhynchoid sawfishes, however, have not been reported from the Weddellian Bioprovince up to now, which indicates that the Weddellian influence on the Austral Basin during the Cenomanian might have been only minor or completely absent. This also would imply that the faunal differentiations related to oceanic currents might have occurred after the Cenomanian. More material from the Cenomanian from northern and southern Patagonia is necessary to establish such patterns in detail. Migration of *Ptychotrygon* to southern South America was facilitated by the successive opening of the southern Atlantic. Whether *Ptychotrygon* was able to establish itself as a successful element of Late Cretaceous South American elasmobranch faunas remains unanswered currently due to the lack of material, which in Patagonia clearly is biased towards large-toothed taxa. More targeted bulk sampling of Cenomanian to Maastrichtian deposits of Patagonia to retrieve abundant micro-toothed taxa is mandatory for better understanding evolutionary and diversity patterns of southern hemisphere elasmobranchs.
